# Motivation communication training programme for healthcare professionals to support adherence in patients with diabetic foot ulcers: Proof of concept study

**DOI:** 10.1371/journal.pone.0295180

**Published:** 2024-02-08

**Authors:** Jennie E. Hancox, Wendy J. Chaplin, Charlotte E. Hilton, Noemi Vadaszy, Katie Gray, Fran Game, Kavita Vedhara

**Affiliations:** 1 School of Medicine, University of Nottingham, Nottingham, United Kingdom; 2 School of Sport, Exercise and Health Sciences, Loughborough University, Loughborough, United Kingdom; 3 Department of Health Sciences, University of Leicester, Leicester, United Kingdom; 4 Derbyshire Community Health Services NHS, Bakewell, United Kingdom; 5 University Hospitals of Derby and Burton NHS Foundation Trust, Derby, United Kingdom; USC Keck School of Medicine: University of Southern California Keck School of Medicine, UNITED STATES

## Abstract

Patients with diabetic foot ulcers have poor adherence to treatment recommendations. However, the most effective way to support adherence in this population is unknown. This study aimed to assess the preliminary effectiveness of a motivation communication training programme for healthcare professionals working with these patients, using theory and evidence-based strategies.A proof-of-concept study using a non-randomised, controlled before-and-after design. Six podiatrists took part in the motivation communication training programme. Pre-training, observation was undertaken to examine the communication style currently used by podiatrists in routine consultations. Patients’ (n = 25) perceptions of podiatrist autonomy support, self-determination for limiting weight-bearing activity and average daily step count were also assessed. Post training, observations and patient measures were repeated with a different group of patients (n = 24). Observations indicated that podiatrists exhibited a more need-supportive communication style (e.g., taking time to understand patients’ perspectives) after undergoing the training programme. Patients in the post-training group reported higher levels of autonomy support, while self-determination to limit weight-bearing activity remained unchanged. Although the post-training group had a lower average daily step count, the difference was not statistically significant. This is the first study to investigate implementation of motivation communication strategies in routine consultations with patients with diabetic foot ulcers. Results suggest that training can enhance healthcare professionals’ motivation communication skills with potential for addressing adherence issues, however, a larger cluster randomised controlled trial is necessary to confirm this.

## Introduction

Diabetic foot ulcers (DFUs) are a chronic complication of diabetes characterised by lesions in the skin of the lower limbs. Estimated to effect about 6.4% of the 463 million adults with diabetes worldwide, DFUs are a serious public health problem [1]. DFUs can negatively affect physical and psychosocial functioning and are associated with a high incidence of lower limb amputation and mortality [2–4]. The financial impact is substantial with annual costs estimated as £837-£962 million in the UK alone [5].

Following recommended treatments (e.g., limiting weight-bearing activity, wearing pressure-relieving therapeutic footwear) is crucial for ulcer prevention and healing [6,7]. Yet, patients with DFUs often struggle to comply [8–10]. Adherent patients have significantly better outcomes than those who are non-adherent [9]. Thus, the International Working Group of the Diabetic Foot have advocated for prioritisation of adherence in patient communication and advised that development, evaluation and implementation of new interventions addressing the adherence problem are urgently needed [11].

Effective physician-patient communication is vital for adherence [12]. Patients living with DFUs have reported dissatisfaction with patient-provider communication [13–15], due to directive and generic treatment advice, lack of empathy and confusion in how to appropriately implement treatment advice [13–15]. To address these issues, we have developed a motivation communication training programme for healthcare professionals focussed on facilitating discussions around motivation and adherence to treatment recommendations in patients with DFUs. Development and acceptability of the training programme have been described elsewhere [16]. In brief, the training involved 6 x 1-hour face-to-face sessions delivered over an 8-week period and was underpinned by Self-determination theory (SDT) [17] and Motivational Interviewing (MI) [18].

MI is a collaborative conversation approach to promoting behaviour change which uses techniques (e.g., open questions, affirmations, summaries) to help individuals identify their motivations and values. SDT is a theory of motivation that focuses on satisfaction of individuals’ basic psychological needs for autonomy, competence and relatedness to promote self-determined motivation (i.e., engaging in a behaviour because it is based on ones’ own values, interests or goals, rather than external pressures). Self-determined motivation is associated with more adaptive health outcomes, better behavioural adoption and long-term maintenance [17,19]. According to SDT, when healthcare professionals use autonomy supportive communication (acknowledging values and goals, offering choices, and considering others perspectives) they better fulfil individuals’ basic psychological needs. MI and SDT are complementary, with SDT providing the theoretical framework for understanding how MI techniques support behaviour change [20]. However, no research has yet explored the integration of SDT and MI within an intervention with health professionals working with patients with diabetic foot ulcers.

Patients with neuropathic DFUs in weight-bearing areas are advised to rest and limit weight-bearing activity [21]. This practice is based on recommendations from the International Working Group of the Diabetic Foot [11], derived from research evidence that patient activity levels are negatively associated with DFU wound healing [22,23]. However, patients have reported limiting weight-bearing activity challenging [14,15]. Thus, exploration as to how to support adherence to support weight-bearing activity reduction in patients with DFUs is needed.

This study aimed to examine preliminary effectiveness of a SDT and MI-based motivation communication training programme for healthcare professionals to support adherence in patients with diabetic foot ulcers. Diabetes-specialist podiatrists play a central role in patient care. They are uniquely placed with the opportunity to engage in behaviour change-related discussions whilst delivering footcare [24], but have expressed frustration in their attempts to guide and build partnerships with patients [25]. The present research addresses this gap by testing the training programme with diabetes-specialist podiatrists to enhance patient interactions and treatment adherence.

More specifically we examined:

- The extent to which the training programme led to changes in podiatrist communication style in routine consultations. It was hypothesised that podiatrists would exhibit a more autonomy supportive communication style following participation in the training programme and that this would be perceived by patients.
- The extent to which the training programme influenced patients’ self-determined motivation to limit weight-bearing activity and adherence to treatment recommendations (i.e., limiting weight-bearing activity). Based on self-determination theory, it was hypothesised that patients seen by podiatrists who had received the communication training programme would report more self-determined motivation and have lower levels of weight-bearing activity compared to patients seen by a podiatrist who had not undertaken the training programme.

## Methods

### Design

A proof of concept study using a non-randomised, controlled before-and-after design. The study was approved by the East Midlands—Derby Research Ethics Committee

(REC Number 18/EM/0162). The study was registered on clinicaltrials.gov (NCT03853941).

### Participants

#### Healthcare professionals

Participants comprised an opportunistic sample of diabetes specialist podiatrists recruited from a specialist Diabetes Foot Clinic in the East Midlands, UK. Podiatrists are primarily responsible for imparting advice regarding limiting weight-bearing activity to patients thus they were considered the most appropriate healthcare professionals to trial the training programme with. Podiatrists were provided with an information sheet which informed them of all aspects pertaining to participation and written informed consent gained. Podiatrists were included if they were working in the specialist Diabetes Foot Clinic, aged 18 and over, had at least 6 months experience working within the NHS.

#### Patients

Patient participants were an opportunistic sample of patients recruited from a specialist Diabetes Foot Clinic in the East Midlands, UK. To take part patients had to be aged 18 years and over, have diabetes according to the World Health Organisation criteria, have a current diabetic foot ulcer and be able to communicate and complete questionnaire measures in English. Patients who were not currently engaging in walking behaviour (e.g., wheelchair users) or who had other physical limitations that restricted their ability to use an activity monitor were excluded. All patients meeting the inclusion criteria were approached and invited to participate in the study.

### Outcome measures

Demographic data were collected for both patients (e.g., age, gender, marital status, ethnicity, number of months attending clinic & diabetes type) and podiatrists (e.g., age, gender, ethnicity, years working for the NHS and years in current role). Patient data from medical records (i.e., number of months attending clinic & diabetes type) were collected between April and December 2019.

#### Primary outcome

*Patients’ perceptions of autonomy support*. Measured using the 6-item Health Care Climate Questionnaire [26]. The stem “Thinking about your visit as a whole today please rate the extent to which you felt that…” preceded items (e.g. ‘My healthcare providers conveyed confidence in my ability to make changes regarding limiting my weight-bearing activity,’). Patients rated items on a 7-point Likert scale (1 = not at all true, 7 = very true).

#### Secondary outcomes

*Patient self-determined motivation*. Measured using the 15-item Treatment Self-regulation Questionnaire [24]. Patients were asked to rate on a scale (1 = not at all true, 7 = very true) the reasons why they would adhere to limiting weight-bearing activity. There are 3 subscales: 8-items assess patients’ autonomous motivation (e.g., The reason I would limit my weight-bearing activity is because I personally believe it is the best thing for my health), 8-items measure controlled motivations (e.g., Because others would be upset with me if I did not), and 3-items amotivation (e.g., I really don’t think about it). Average scores were calculated for each subscale and a Relative Autonomy Index (RAI) formed by subtracting the average for the controlled reasons from the average for the autonomous reasons.

*Patient weight-bearing activity*. Step-count was used as a measure of patient weight-bearing activity [27]. Patient participants were given a triaxial accelerometer (GENEActiv Original, Activinsights, UK) to objectively measure activity levels for a period of 28 days. The GENEActiv has been shown to provide valid estimates of physical activity and sedentary behaviours [28]. Patients were asked to wear the accelerometers on their non-dominant wrist for their convenience. Accelerometer monitors were calibrated for age, height, and handedness and set to capture and store accelerations at the sampling frequency of 25 Hz. Activity data were downloaded using the GENEActiv PC Software (Version 3.2) as raw.bin files.

*Fidelity of delivery*. Observations of consultations were undertaken to assess the extent to which the podiatrists put the motivationally supportive communication strategies into practice as intended. Consultations were live-coded as audio-recording was not possible in the busy clinic environment due to concern over privacy of other nearby patients. Observations were conducted by a trained researcher (WJC) not involved in delivering the intervention training using the BECCI, a tool that can be used to assess the extent to which practitioners exhibit motivational interviewing consistent techniques in brief consultations in healthcare settings [29]. The BECCI has been found to demonstrate acceptable levels of reliability and validity [30]. The BECCI has 11 items, grouped into 4 domains: agenda setting and permission seeking (i.e., the patient is given choice in what issues they would like to discuss in the session and the practitioner explicitly asks the patient’s permission before talking about limiting weight-bearing), the why and how of change in behaviour (i.e., takes time to understand the patient’s perspective using open questions, empathetic listening, and summaries), whole consultation (i.e., acknowledges patient’s perspective, uses affirmations, and does not put pressure on patient to change their behaviour) and talk about targets (i.e., works with the patient to identify barriers, problem solve and set goals). Mean scores were calculated for BECCI individual items, domains and an overall total ‘BECCI Score’ (by summing item mean scores).

Three questions were designed specifically for this study to assess the extent to which the observer perceived the strategies exhibited by the podiatrist to be delivered in a way which supports patients’ basic psychological needs (BPNs) for autonomy, competence and relatedness. All items were rated on a 5-point Likert Scale (0 = not at all, 1 = minimally, 2 = to some extent, 3 = a good deal, 4 = a great extent). Mean scores were calculated for all three items, along with a total ‘BPN Score’ (by summing item mean scores).

Prior to data collection, the observer practiced using the modified BECCI to score six video-recordings of behaviour change consultations [31]. Proficiency was evaluated by comparing scores with two experienced coders (JEH & CEH) to ensure a certain level of consistency (i.e., to score within one point of each other) before undertaking clinic observations.

### Procedure

**Fig 1:**
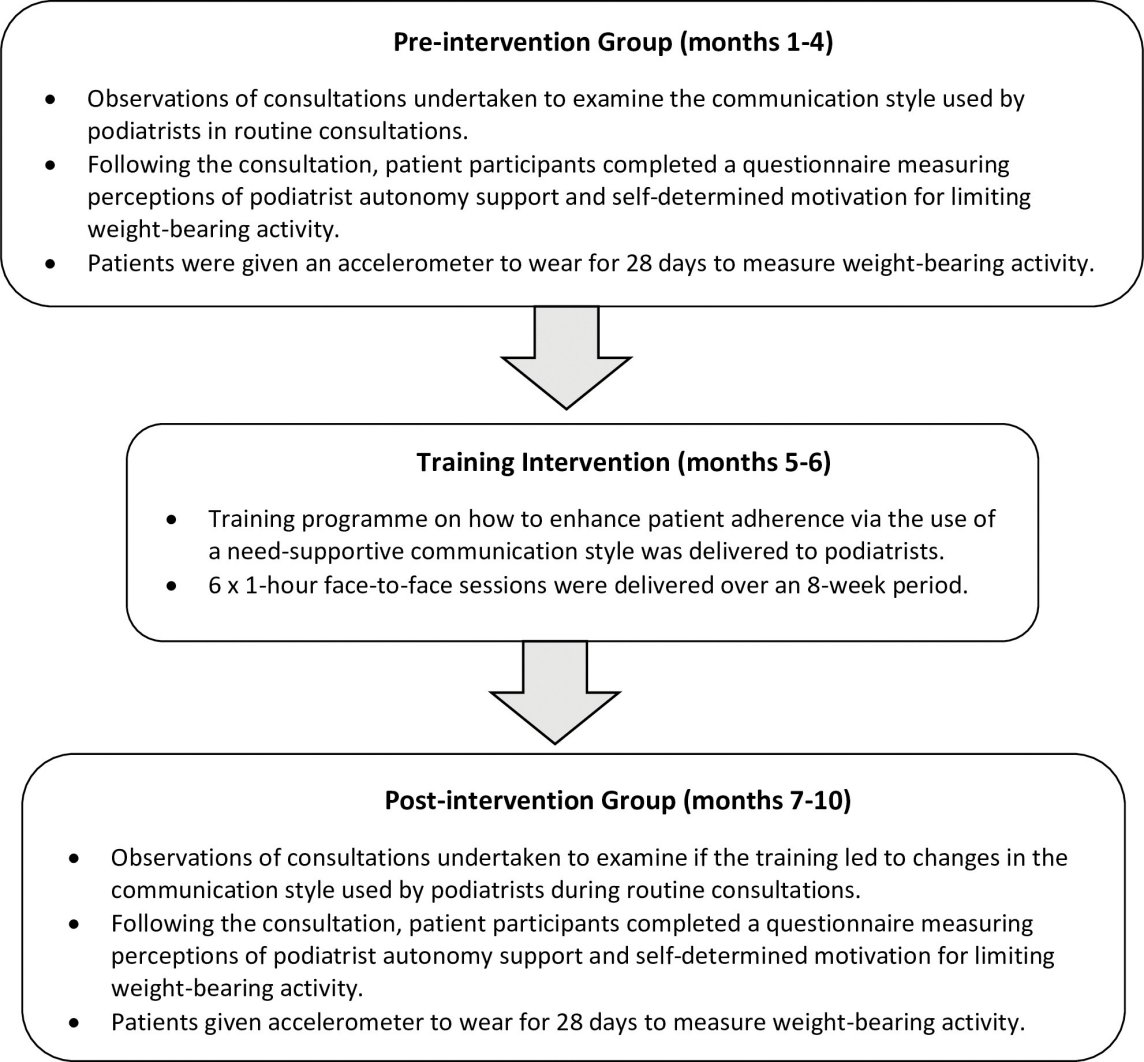
Overview of study procedure.

Fig 1 outlines the study procedure. Eligible patients were initially approached by a member of their usual care team (April-December 2019) and informed of all aspects pertaining to study participation. Written informed consent was gained from all patients expressing a wish to take part. Data was anonymised with each participant assigned a trial identity number for use on study documents and the electronic database. Patients recruited in the first 4 months of the study were assigned to the pre-intervention group. Podiatrists underwent a 6-week motivation communication training programme. Patients recruited after completion of the training programme were assigned to the post-intervention group. No patients were in both the pre- and post-intervention groups. It was not possible to have the same group of patients tested before and after the training period because patients may have been discharged from treatment during the study.

The same procedure was followed for both the pre- and post-intervention groups. Following consent, a researcher observed the patient’s next treatment session. After the session, the patient completed a questionnaire measuring their perceptions of podiatrist autonomy support and motivation for limiting weight-bearing activity. Patients were also given an accelerometer and asked to wear it for 28 days consecutive days. Patients returned the accelerometer using a pre-paid envelope or by handing it to a member of the team at the foot clinic.

### Intervention

We developed an intervention to support patients with DFUs to limit their weight-bearing activity. Details of development of the intervention are provided elsewhere [16]. In brief, diabetes specialist podiatrists received six 1-hour face-to-face training sessions. The training was delivered by two researchers, one experienced in delivering SDT interventions (JEH), the other experienced in delivering MI training to healthcare professionals (CEH). Podiatrists were taught SDT-based motivation communication strategies (see Table 1) relevant to the specific context of a diabetic foot consultation. Strategies were selected from those used in previous SDT interventions [32,33] and similarly to other SDT applied research [34,35], MI techniques (e.g., open questions, reflections) were included as a means of promoting basic psychological need satisfaction.

**Table 1:** Motivation strategies organised by MI process, adapted with permission from Hancox et al. [16].

MI process	Aim of process	SDT-based strategy	Description of strategy	Basic need(s) targeted	Training Session
Engaging (to be maintained throughout the consult)	Develop rapport, empathy and take time to listen to and understand the patient’s perspective	Use non-controlling language	Use language that emphasises the patient’s right to choose and avoid the ‘*righting reflex*’ (i.e., telling patients what they should do)	Autonomy	1
		Develop involvement by demonstrating warmth and empathy	Express a personal interest in the patient and take time to develop a rapport. Use *open-ended questions* and *reflective listening* statements.	Relatedness	2
		Acknowledge patient’s perspectives	Take time to understand the patient’s perspective and recognise their challenges. Use *affirmations* that acknowledge the patient’s difficulties, efforts and self-worth.	Autonomy	3
Focusing (What?)	Establish personal context and factors relevant to the patient’s experience of their DFU and limiting weight-bearing	Offer choices	Acknowledge the patient’s ability for choice and self-determination. Ask about the patient’s concerns and priorities and what they would like to focus on (shared *agenda setting*).	Autonomy	4
		Take time to understand the patient’s personal context and factors relevant to the target behaviour	Invite the patient to talk about their day-to-day life and how relevant and practical limiting weight-bearing is for them. Use the *typical day* technique (e.g., “Talk me through a typical day for you but with a focus upon when you might be at your most active”).	Autonomy & relatedness	4
Evoking (Why?)	Explore the patients’ personal interest and motivation to limit activity & weight- bearing	Explore patient’s reasons for changing behaviour	Explore the patient’s reasons for limiting weight-bearing or not. Use *scaling questions to assess importance* (e.g., “On a scale of 1–10, how important is it for you to limit your activity and weightbearing?” and *open-ended questions* that seek to elicit change talk (e.g., “Why are you a 5 and not a 3?”, “What needs to happen for you to get to a 6?”).	Autonomy	5
		Explore patient’s values relating to the target behaviour	Explore patient’s values and how they relate to target behaviour. Use the *‘two possible futures’* technique and invite patients to imagine what their life might be like if their ulcer did or did not heal in the future and describe what that might mean for them.	Autonomy	5
		Support the patient with barrier identification and problem solving	Work with the patient to identify barriers to behaviour change. This may include the use of *scaling questions to assess confidence* to limit-weight-bearing (e.g., “On a scale of 1–10, how confident are you that you can limit your activity and weight bearing?”, “Why are you a 5 and not a 3?”, “What needs to happen for you to get to a 6?”) and problem solving.	Competence	5
		Provide information and rationales	Provide information and rationales relevant to the patient’s needs and situation (e.g., about antecedents or health consequences of the behaviour). Use the technique ‘*Elicit-Provide-Elicit*’ to: 1) Elicit what the patient knows or would like to know or if it’s okay if you offer them information, 2) Provide the information in a neutral, non-judgmental fashion, and 3) Elicit the patient’s interpretation.	Autonomy	5
Planning (How?)	Develop a plan to limit weight-bearing that is specific, detailed & individualised	Provide structure	Set parameters within which choice and agency can take place and provide support to initiate action. This may involve developing an appropriate individualised plan according to the patient’s specific context and needs. Techniques may include: jointly agreeing SMART goals, action planning (e.g., if…then plans) and *summaries* (e.g., verbally summarise the conversation and provide a written summary for the patient to take home with them).	Autonomy & Competence	6

Note. MI techniques are provided in italics.

The training was delivered over an 8-week period (first 4 sessions weekly, and last 2 sessions biweekly) to enable podiatrists’ time to practice using the communication strategies between sessions. Details of which strategies were covered in which session are outlined in Table 1. The training content was structured based upon the four processes of MI: engaging, focusing, evoking and planning [18].

A mix of PowerPoint slides, video examples, small group discussions and role-play activities were used to support learning. Podiatrists were encouraged to practice implementing the strategies in routine consultations, share experiences and problem solve together. A written summary of the practical strategies and an audio recorded summary of the key points covered in each training session were provided. Podiatrists were advised to use the audio recording as a reminder of what was covered and as a means of catching-up for those unable to attend any sessions.

### Data analysis

Quantitative data analysis was undertaken using SPSS (version 26). Descriptive statistics (means, standard deviations, range for continuous data and frequencies and percentages for categorical data) were calculated for demographic characteristics of patient participants and healthcare professionals and questionnaire measures. For analysis of accelerometer data Raw.bin files were converted into.exe and visual.doc files via the ‘R Markdown’ package within the R studio software (https://www.r-project.org/) to calculate step count. To be included in the analysis, the participants had to wear the accelerometer for at least 7 days and at least 7h/day of valid wear time. Average daily step count was calculated for each individual using all available data. Independent *t*-tests were used to explore differences between study arms in patient perceptions of podiatrist autonomy support, treatment self-regulation, mean daily step count and observation scores.

## Results

**Fig 2:**
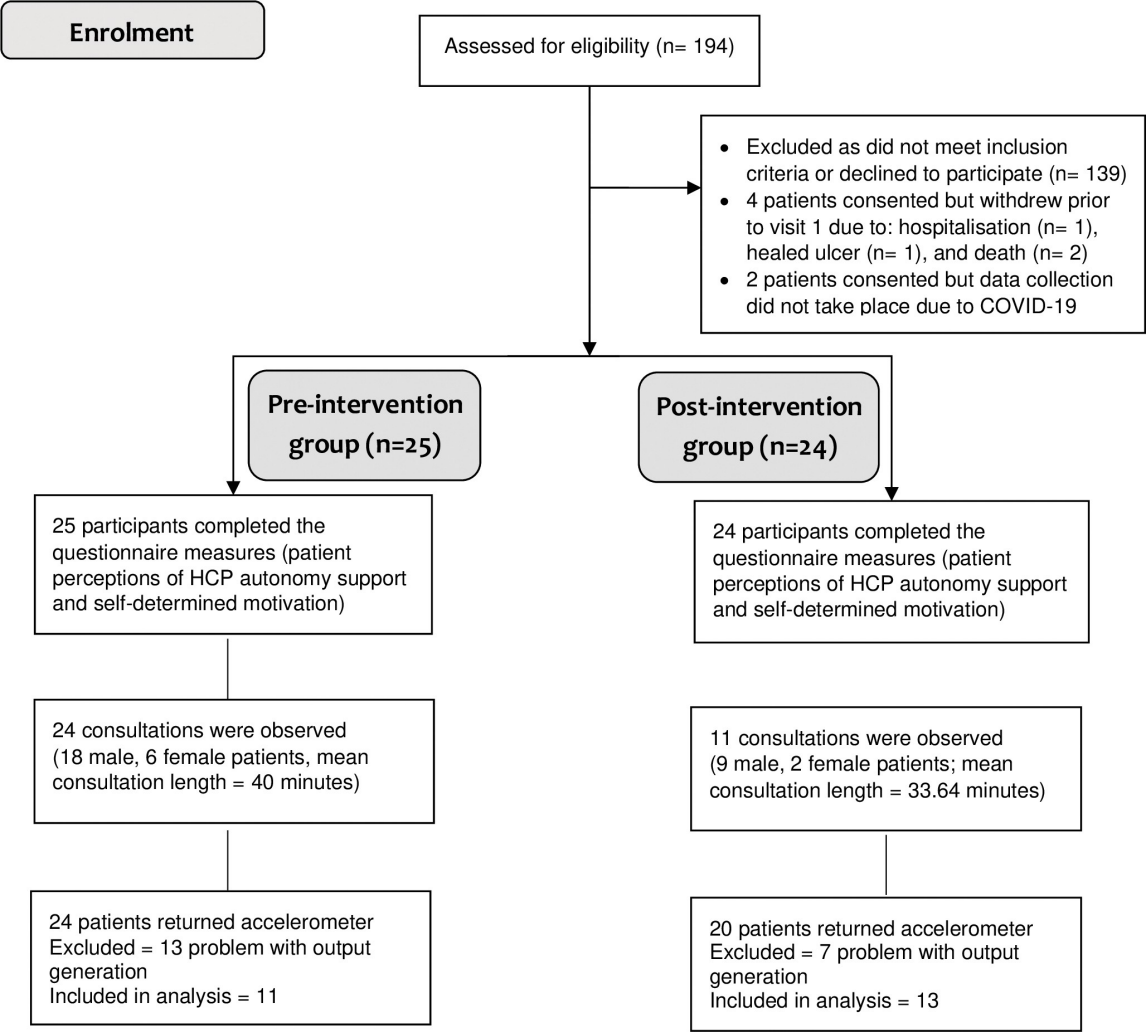
CONSORT diagram.

The CONSORT diagram (Fig 2) summarises the screening and recruitment of patient participants including how many took part in the different study components (i.e., observations, activity monitoring). Demographic and clinical characteristics of the pre-intervention (n = 25) and post-intervention groups (n = 24) are displayed in Table 2.

**Table 2:** Demographic characteristics of patient participants.

	Pre-intervention (n = 25)	Post-intervention (n = 24)
Age in years		
Mean (SD)	60.72 (11.02)	58.42 (11.50)
Range	35–81	28–81
Gender–N (%)		
Male	20 (80%)	21 (87.5%)
Female	5 (20%)	3 (12.5%)
Marital status–N (%)		
Single	7 (28%)	8 (33.3%)
In a relationship	1 (4%)	3 (12.5%)
Married	12 (48%)	12 (50%)
Divorced	2 (8.0%)	0 (0%)
Widowed	3 (12%)	1 (4.2%)
Ethnicity–N (%)		
White British	25 (100%)	24 (100%)
Body mass index		
Mean (SD)	31.56 (6.54)	35.76 (7.81)
Range	22.53–46.92	25.57–50.76
Number of months patient had been attending clinic		
Mean (SD)	16.28 (12.33)	19.46 (20.95)
Range	0–34	1–72
Type 1 diabetes–N (%)	6 (24%)	2 (8.3%)
Type 2 diabetes–N (%)	19 (76%)	22 (91.7%)

Six podiatrists (1 male, 5 female; mean age = 35.83, SD = 11.41, all White British) took part in the training programme. On average podiatrists had worked in the NHS for 9 years (range = 4–17 years) and had been in their current role for 5 and half years (range = 1–17 years). Three podiatrists attended all six training sessions (100%). One podiatrist attended 5/6 sessions (83%) and two attended 4/6 sessions (67%). Those that missed a session were encouraged to listen to the provided audio recorded summary.

### To what extent did the training programme lead to changes in podiatrist communication style?

Patient perceptions of podiatrist autonomy support was higher in the post-intervention group compared to the pre-intervention group (see Table 3 for details). The difference was statistically significant (p = 0.04) with medium effect size (*d* = -.62).

**Table 3:** Outcome measure scores for pre-intervention and post-intervention groups.

Measure	Pre-intervention Mean (SD)	Post-intervention Mean (SD)	T-test
Autonomy support (α = .94)	5.58 (1.73)	6.42 (0.77)	t(33) = -2.20, p = 0.04, *d* = -.62, 95% CI [-1.19, -0.04]
Motivation for limiting weight-bearing			
Autonomous (α = .90)	5.74 (1.33)	5.72 (1.47)	t(45) = 0.05, p = 0.96, *d* = .01, 95% CI [-0.56, 0.59]
Controlled (α = .87)	4.08 (1.84)	3.92 (1.96)	t(43) = 0.28, p = 0.79, *d* = .08, 95% CI [-0.50, 0.67]
Amotivation (α = .55)	2.88 (1.63)	2.89 (1.55)	t(46) = -0.03, p = 0.98, *d* = -.01, 95% CI [-0.57, 0.56]
RAI	1.60 (1.69)	1.63 (1.80)	t(41) = -0.07, p = 0.95, *d* = -.02, 95% CI [-0.62, 0.58]
Step count/activity data	4390.36 (2585.07)	2694.78 (1928.51)	t(22) = 1.84, p = 0.79, *d* = .75, 95% CI [-0.87; 1.58]

Table 4 displays BECCI individual item, domain, and total observation scores for both the pre-intervention and post-intervention patient groups. Observations revealed podiatrist use of MI-informed behaviour change counselling techniques (BECCI total score) to be significantly higher post- compared to pre-intervention, with a large effect size (*d* = -3.50). Post-training, podiatrists were observed to be more likely to invite patients to talk about behaviour change, this includes explicitly asking the patient permission to talk about behaviour change. The greatest change was in the domain ‘The Why and How of Change in Behaviour’ which involved taking time to understand the patient’s perspective using techniques such as open questions, reflective listening, and summaries. There was less change in the domains ‘The whole conversation’ which involves acknowledging challenges and respecting patient choice regarding behaviour change and ‘Talk about Targets’ which includes working with the patient to identify barriers, problem solve and set goals.

**Table 4:** Observation results: Individual item, domain and total BECCI scores (mean, SD, *t*-test).

		Item scores		Domain scores		T-tests
Domain	Item	Pre-intervention	Post- intervention	Pre- intervention	Post- intervention	
		Mean (SD)	Mean (SD)	Mean (SD)	Mean (SD)	
1. Agenda setting and permission seeking	1. The patient invites the practitioner to talk about behaviour change	0.65 (0.41)	2.82 (0.87)	0.91 (0.46)	2.64 (0.60)	t(33) = -9.38, p = 0.00, *d* = -3.42, 95% CI [-4.49; -2.32]
	2. The practitioner demonstrates sensitivity to talking about other issues	1.17 (0.64)	2.45 (0.69)			
2. The why and how of change in behaviour	3. Practitioner encourages patient to talk about current behaviour or status quo	1.17 (0.87)	2.73 (0.47)	0.56 (0.40)	2.36 (0.62)	t(33) = -10.45, p = 0.00, *d* = -3.81, 95% CI [-4.96; -2.64]
	4. Practitioner encourages patient to talk about behaviour change	0.38 (0.58)	2.55 (1.04)			
	5. Practitioner asks questions to elicit how patient thinks and feels about the topic	0.63 (0.71)	2.55 (0.69)			
	6. Practitioner uses empathic listening statements when patient talks about the topic	0.46 (0.51)	2.45 (0.82)			
	7. Practitioner uses summaries to bring together what the patient says about the topic	0.17 (0.48)	1.55 (1.04)			
3. The whole conversation	8. Practitioner acknowledges challenges about behaviour change that the patient faces	1.13 (0.85)	2.64 (0.92)	1.02 (0.65)	2.61 (0.88)	t(33) = -5.97, p = 0.00, *d* = -2.18, 95% CI [-3.05; -1.28]
	9. When practitioner provides information, it is sensitive to patient concerns and understanding	1.15 (0.64)	2.55 (0.93)			
	10. Practitioner actively conveys respect for patient choice about behaviour change.	0.79 (0.78)	2.64 (1.03)			
4. Talk about targets	11. Practitioner and patient exchange ideas about how the patient could change current behaviour	0.77 (0.53)	1.94 (0.52)	0.77 (0.53)	1.94 (0.52)	t(33) = -6.12, p = 0.00, *d* = -2.23, 95% CI [-3.11; -1.28]
TOTAL BECCI SCORE		8.37 (4.45)	26.85 (6.83)			t(33) = -9.59, p = 0.00, *d* = -3.50, 95% CI [-4.59; -1.30]

N.B. Each item was rated on a five-point Likert scale (0 = not at all to 4 = a great extent).

Individual item and total observation scores for basic psychological needs satisfaction are presented in Table 5. Consultations delivered by podiatrists who attended the motivation communication training programme were perceived to be delivered in a more need-supportive way (BPNS total score), with a significant difference between groups (t(33) = -5.44, p = 0.00, *d* = -1.98, 95% CI [-2.83; -1.11]). Perceived feelings of relatedness were observed to be higher than support for autonomy and competence both before and after the training.

**Table 5:** Observation results: Individual item and total scores for basic psychological needs satisfaction (mean, SD).

		Mean (SD)	
		Pre-intervention	Post-intervention
BPNS	Autonomy	1.2	2.6
	Competence	1.2	2.5
	Relatedness	1.8	3.2
	TOTAL SCORE	4.13 (2.11)	8.27 (2.05)

*BPNS–basic psychological need satisfaction.

### To what extent did the training programme influence patients’ self-determined motivation and weight-bearing activity?

Descriptive statistics (Mean, SD) and comparisons by group for measures of patient motivation and step count are presented in Table 3. Patients generally reported higher levels of autonomous motivation for limiting weight-bearing activity and lower levels of controlled motivation. Mean levels of controlled motivation were slightly lower in post- compared to pre-intervention patient participants. However, differences between groups in patient self-reported motivation for limiting weight-bearing activity were not significant. Average daily step count was lower for patients seen by podiatrists who had attended the communication training programme, with a medium effect size (*d* = .75).

## Discussion

To our knowledge, this is the first study to explore integration of SDT-based motivation communication strategies into routine consultations with patients living with DFUs. Findings revealed patients to perceive podiatrists to become more autonomy supportive in consultations following the training programme. These results are aligned with SDT-based interventions in other health contexts which have found health professionals trained to become more autonomy supportive to exhibit greater autonomy support post-training compared to a control group [19,33]. Despite being on the front line of patient care, podiatrists currently receive little or no training in how to support behaviour change effectively during regular routine consultations. Findings suggest the training programme may hold promise for addressing patients reported dissatisfaction with patient-provider communication [13,14] and promoting a more motivationally supportive approach.

Podiatrists effectively utilised motivational techniques taught in training during routine consultations. However, there was less emphasis on the later stages of MI (i.e., evoking and planning processes) which involve identifying barriers and setting goals. This may be due to the training prioritising the initial MI processes, ensuring adequate time was spent engaging and focusing to avoid the premature focus trap [18]. Additionally, some patient-centred conversations may not progress to evoking and planning within the first few sessions due to participant readiness. Longer observations of consultations would provide greater understanding of how adherence conversations are continued over time.

To effectively support adherence to treatment recommendations, it is important to understand patients’ motivation. Patients were found to be more autonomously motivated to limit weight-bearing activity (i.e., they believe it is important for their health). An interview study exploring patients’ experience of being advised to limit weight-bearing activity described providing a rationale, such as the health benefits, as a key facilitator to adherence [15]. The findings from the current study add to this knowledge in terms of emphasising that doing so in a more person-centred and MI-informed way, such as by asking permission using the ‘Elicit-Provide-Elicit’ technique, may further influence patient behaviour change.

Levels of controlled motivation, although moderate, suggests some patients were motivated by external factors such as, avoiding letting oneself-or others down, or feeling pressured by significant others. Although non-significant, controlled motivation was lower in patients in the post-intervention group indicating podiatrists trained in SDT- and MI-based communication skills may have reduced their use of controlling language (e.g., “you need to limiting weight-bearing”). However, patients’ perceptions of podiatrist controlling behaviours were not measured in this study, doing so in future research would further enhance understanding of the potential benefits of the motivation communication training.

### Strengths and limitations

This study makes an important contribution to the literature by advancing understanding of the practicalities of translating motivational principles into practice in routine consultations with patients. A strength of the study was the combination of MI communication techniques and SDT theoretical constructs. Integration of the complementary approaches can strengthen both practice and understanding of the processes underlying behaviour change [20].

A novel aspect of the study was the use of live-coded observation using the BECCI [29] to explore the extent to which podiatrists put the motivation strategies into practice. Audio-recording of medical consultations are not always possible due to privacy concerns; therefore live-coding can provide an alternative means of assessing fidelity of delivery of motivation strategies with difficult to reach populations. However, presence of an observer within consultations may increase the risk of social desirability bias, with healthcare professionals temporarily increasing their use of the taught motivation strategies.

It is important to note that this proof-of-concept study was not powered to detect significant changes in study measures therefore we are only able to provide preliminary indication as to effectiveness of the intervention. A future cluster randomized controlled trial is required to establish the feasibility and effectiveness of the intervention more widely. In such a future trial it would be beneficial to collect more information regarding participant demographics (e.g., education level, socioeconomic status) and medical history (e.g., type of ulcer, history of amputation, time with ulcer and other health conditions) to explore the extent to which these factors may influence motivation for treatment adherence.

Further limitations of the study include a small sample size, being conducted at only one diabetic foot clinic in the UK, and podiatrists’ communication style being assessed only once post-training. Assessment of podiatrists’ use of motivation strategies at multiple time-points would determine if the effects of the intervention persist over time. Future research is needed to explore applicability and acceptability of the training programme for other diabetes-specialist healthcare professionals.

## Conclusion

The study demonstrates the effectiveness of the motivation communication training programme in altering healthcare professionals’ communication style. Following the training, podiatrists were perceived to adopt a more autonomy supportive approach and employ more MI-informed behaviour change counselling techniques. Thus, the programme holds promise for addressing patient dissatisfaction with the delivery of treatment advice and improving patient adherence.
